# 80 Case series of juvenile scleroderma at Aga Khan University Hospital, Nairobi

**DOI:** 10.1093/rheumatology/keac496.076

**Published:** 2022-10-07

**Authors:** Irene Nzisa, Chemutai Mercy, Angela Migowa

**Affiliations:** Department of Pediatrics and Child Health, Aga Khan University Medical College East Africa, Nairobi

## Abstract

**Background:**

Juvenile scleroderma includes a range of conditions presenting with skin fibrosis. It’s classified into two major categories, localized and systemic. Juvenile localized scleroderma (morphea) is limited to the skin and forms majority of pediatric presentations (>95%). Though rare, juvenile systemic sclerosis involves the skin and other internal organs and is associated with a poorer prognosis.

**Objective:**

To characterize the clinical profile of two cases of scleroderma at Aga Khan university hospital, Nairobi.

**Methods:**

Retrospective review of medical records of two patients with Juvenile scleroderma managed at Aga Khan university hospital, Nairobi.

**Results:**

The first patient was a 7-year-old boy presenting with joint pains for 2 months, restricted activities and limping. He also had a non-itchy lesion on the right thigh for about 6 years. On examination, there was an 8 by 18 cm, flat, shiny skin lesion with ill-defined margins and skin thickening around it. There were 2 punched out scars from previous skin biopsy sites with latest biopsy showing atrophic epidermis, dermal collagen homogenization with thick sclerosed fibers in keeping with morphea. Pulmonary function tests revealed mild restrictive lung disease. There were no other systems involved on further evaluation. He was started on weekly methotrexate, daily prednisone, daily folic acid and omeprazole to be followed up in clinic.

The second case is a 5-year-old girl who presented with 3-year history of inability to walk. Physical examination revealed areas of skin hypopigmentation and hyperpigmentation on the neck and back with active lesions on the left flank and posterior right leg. She had left lower limb atrophy and extreme wasting of thigh and calf muscles, a contracture of the left knee, ankle and limb length discrepancy. Her investigations revealed positive rheumatoid factor, positive antinuclear antibody (ANA), microcytic hypochromic anaemia and skin biopsy with features of morphea. She received six courses of high dose methyl prednisone over six months, weekly methotrexate, daily folic acid, daily prednisone that was tapered off gradually, calcium supplementation and iron supplementation. In addition, she received isoniazid for tuberculosis prophylaxis with pyridoxine. Her management was multidisciplinary involving orthopedics, physiotherapy, occupational therapy, and dieticians. She developed a new skin lesion on the left forearm about five months after initiation of treatment but no worsening of symptoms or other new lesions thereafter.

**Conclusion:**

Juvenile scleroderma though rare has a high risk of misdiagnosis with delay in initiation of treatment. It is associated with significant morbidity and early diagnosis, treatment and follow up is imperative for good outcomes.

